# Microstructure Evolution and Performance Improvement of Silicon Carbide Ceramics via Impregnation Method

**DOI:** 10.3390/ma15051717

**Published:** 2022-02-24

**Authors:** Wei Li, Conghui Guo, Congcong Cui, Jianxun Bao, Ge Zhang, Yubei Zhang, Shan Li, Gong Wang

**Affiliations:** 1 Changchun Institute of Optics, Fine Mechanics and Physics, Chinese Academy of Sciences, Changchun 130033, China; liwei199308@126.com (W.L.); cuicongconghit@126.com (C.C.); baojianxun@ciomp.ac.cn (J.B.); zhanggeciomp@126.com (G.Z.); 2 Key Laboratory of Optical System Advanced Manufacturing Technology, Chinese Academy of Sciences, Changchun 130033, China; 3 Technology and Engineering Center for Space Utilization, Chinese Academy of Sciences, Beijing 100094, China; zhangyubei18@csu.ac.cn (Y.Z.); lishan@csu.ac.cn (S.L.); wanggong@csu.ac.cn (G.W.)

**Keywords:** impregnation method, liquid silicon infiltration, microstructure evolution, mechanical properties, SiC ceramics

## Abstract

The high topological silicon carbide (SiC) ceramics can be prepared by stereolithography (SLA) combined with liquid silicon infiltration (LSI) techniques. This paper aims to enhance the performance of SiC ceramics prepared by SLA and LSI techniques via the cyclic impregnation/carbonization of the precursor of carbon source solution before LSI. The effects of impregnation/carbonization cycles on the microstructure and properties of C/SiC preform and sintered body were analyzed in detail. The results show that, with the increase of impregnation/carbonization cycles, the porosity in the C/SiC preform decreases obviously and the content of secondary SiC in the sintered body increases effectively. Especially, when the impregnation/carbonization cycle was performed twice, the sintered body had the optimal mechanical properties. The value of flexural strength, bulk density and elastic modulus were 258.63 ± 8.33 MPa, 2.95 ± 0.02 g/cm^3^ and 425.16 ± 14.15 GPa, respectively. In addition, the thermal dimensional stability of sintered body was also improved by this method. This method proves that SiC ceramics prepared by SLA combined with LSI have the potential of applications in space optical mirrors.

## 1. Introduction

Silicon carbide (SiC) is the main candidate material for spatial optical mirrors, due to its excellent thermal dimensional stability, low density and high strength [1,2,3]. In order to reduce the launch cost and ensure the imaging accuracy, the SiC mirrors should be designed with optimal topological structures to achieve a high degree of lightweight. However, traditional manufacturing techniques faces great challenges in the preparation of topological and lightweight SiC mirror blanks, due to the high strength and hardness of SiC [4,5]. In recent years, additive manufacturing has been widely considered to be a completely new path to prepare the topological SiC ceramics, as it is an effective method to transform virtual 3D models with highly topological structures into real objects [6]. At present, additive manufacturing widely used in the preparation of ceramics includes binder jet printing (BJP) [7], direct ink writing (DIW) [8], selective laser sintering (SLS) [9] and stereolithography (SLA) [10,11]. Among these technologies, SLA has the advantages of being cost-saving and time-saving, as well as high precision. Generally, the green body fabricated by additive manufacturing needs to be sintered to obtain the properties suitable for application. For SiC ceramics, the common sintering techniques include liquid phase sintering [12], hot-pressing sintering [13] and liquid silicon infiltration [14]. Compared with other techniques, liquid silicon infiltration (LSI) has the advantages of a short cycle, near net-shape forming and excellent performance of the sintered body (RB-SiC). Many researchers [10,12,15] successfully fabricated RB-SiC ceramics via SLA combined with LSI. However, the problem of low mechanical properties generally existed. Especially, the flexural strength is lower than 300 MPa, or even 240 MPa [10,12]. Compared with the RB-SiC ceramics prepared by traditional techniques, the properties are insufficient [5]. Therefore, the low mechanical properties are the obstacles to the application of RB-SiC ceramics formed by SLA.

The fundamental research on sintering process, densification method and strength mechanism is of great significance for promoting the application of SLA technique in the field for the preparation of SiC components. Most of the research in this field is aimed at verifying the feasibility of SLA technique for fabricating SiC ceramics with complex shapes. However, there are few research studies on improving the mechanical properties of SiC prepared by SLA and LSI [10,12,15]. In terms of phase composition of the composites, it is necessary to decrease the content of Si, while increasing the content of SiC [16]. One of the most effective methods is to supplement carbon sources in the SiC green body, and then the carbon is converted into SiC via LSI techniques. Zhang [15] mixed carbon fiber with raw SiC powders and prepared SiC green body by using SLA technology, which improved the content of carbon, and the RB-SiC obtained by LSI technology gave an excellent performance. However, carbon fiber is expensive, and this increases the cost of the preparation process. Xu [17] prepared the porous carbon preform by using phenolic resin (PF) via SLA, and SiC ceramic was obtained after LSI. The results showed that the residual carbon was observed in the final body as the impurity after LSI, due to the fact that the high carbon content in the preform and the impurity would deteriorate the properties of SiC [17]. Fortunately, Ji-Won Oh [7] prepared SiC green body by using BJP and obtained C/SiC preform after debinding. The proportion of SiC in the final body was increased via the cyclic impregnation of PF resolution in C/SiC preform, and the RB-SiC obtained good comprehensive properties [7]. This method is a feasible method to enhance the properties of RB-SiC and the low price of PF saves the cost of preparation [18]. Compared with the idea of adding the extra carbon during SLA, this method is simple to operate and can introduce much carbon into the C/SiC preform. However, this method has hardly been reported to enhance the performance of SiC prepared by SLA and LSI techniques. Meanwhile, the effect of microstructure evolution on the mechanical properties of SiC ceramics prepared by SLA and LSI has not been reported yet.

In this work, SiC was obtained via SLA combined with LSI technology. In order to give the SiC excellent properties, cyclic impregnation/carbonization of PF solution was carried out in C/SiC preform. We aimed to study the effects that the number of times that impregnation/carbonization is performed has on the characteristics of C/SiC preform and RB-SiC. The authors want to provide helpful guidance on improving the properties of SiC prepared by SLA and LSI.

## 2. Materials and Methods

**Figure 1:**
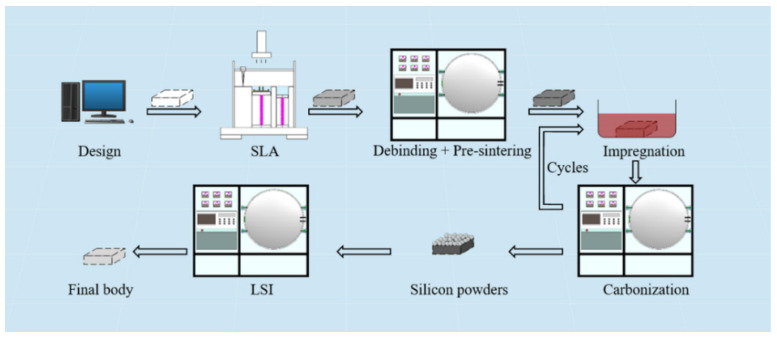
Schematic of fabrication of RB-SiC ceramics with combined SLA, cyclic impregnation/carbonization (0–3 times) and LSI technology.

The experiment process is shown in Figure 1. A 3D model of the target parts was designed via a computer and transferred to a 3D printer. SLA technology was carried out to transform the virtual 3D model into the SiC green body. The SiC green body was composed of raw SiC powders (Weifang Xinfangjing fine Powder Co., Ltd., Weifang, China) and solidified organic matters with a compact surface. The C/SiC preform was obtained after debinding and pre-sintering, and it had high porosity. (i) The C/SiC preform was immersed in the PF solution, and the PF solution entered the C/SiC preform through the pores to realize an impregnation process. Then (ii) the C/SiC impregnated with PF solution was carbonized. Multiple impregnation/carbonization was accomplished by repeating (i) and (ii) processes. Finally, the Si powder was uniformly distributed on C/SiC preform, and RB-SiC was obtained by LSI technology.

(1) For 3D-printing process

**Figure 2:**
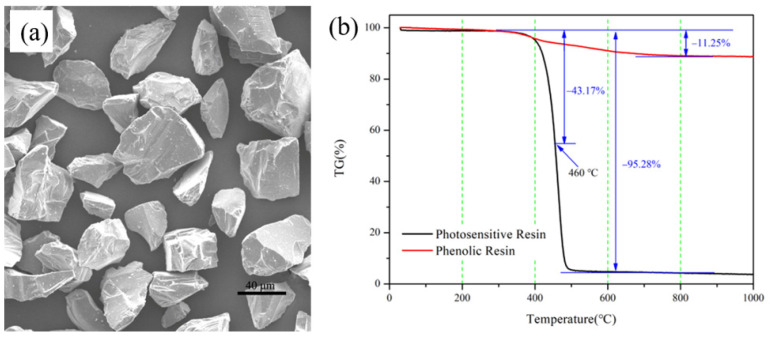
Properties of raw materials: (**a**) the morphology of SiC powder; (**b**) thermogravimetric analysis curve of photosensitive resin and phenolic resin.

The raw SiC powders(Weifang Xinfangjing fine Powder Co., Ltd., Weifang, China), photosensitive resin (Dongguan Inoue new material development Co., Ltd, Dongguan, China) and photoinitiator(Shanghai Aladdin Biochemical Technology Co., Ltd., Shanghai, China) were homogenized by a planetary ball-mill machine(Changsha Miqi Instrument Equipment Co., Ltd., Changsha, China) at the speed of 300 r/min. Five hours later, the SiC ceramic slurry with a solid content of 47 vol.% was obtained. Figure 2 shows the morphology of raw SiC powders and the thermogravimetric (TG) analysis curve of photosensitive resin (PR).

The designed STL format file was transferred to the 3D printer (CeraStation160, Jiangsu qiandugaoke Co., Ltd., Wuxi, China) to start the preparation process, as shown in Figure 1. During the 3D printing process, the UV wavelength, light energy density, exposure time and the thickness per layer were set as 405 nm, 80 μw/cm2, 2 s and 75 μm, respectively. First, the ceramic slurry was scraped by the scraper, and the UV light activates the initiator. Then, the free-radical ions were released by the initiator to promote the crosslinking of PR and a two-dimensional structure was obtained. Finally, the two-dimensional structure was lowered for a layer thickness, and the process was repeated until the end of the program, and the 3D-SiC was obtained.

(2) For debinding and pre-sintering

**Figure 3:**
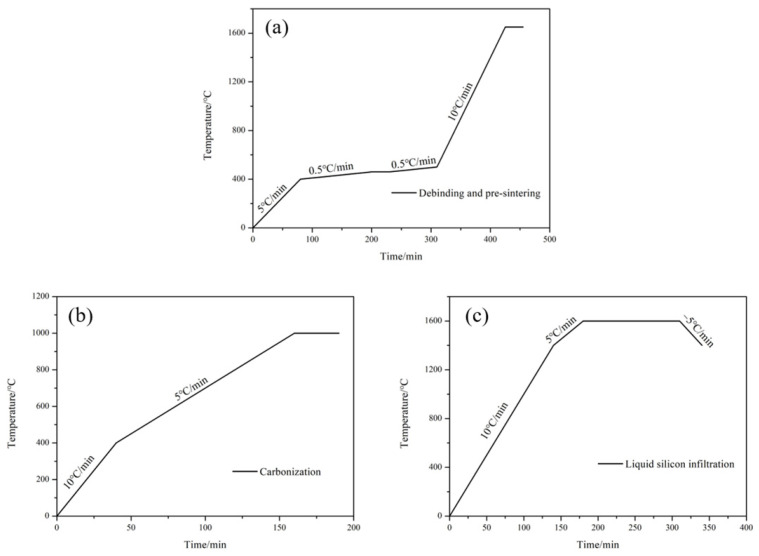
Heating program: (**a**) debinding/pre-sintering, (**b**) carbonization and (**c**) LSI.

The debinding is an essential step in the preparation of SiC ceramics by SLA/LSI method. In this process, it is necessary to control the heating rate and the holding time to avoid the defects, such as cracks and warps, which will deteriorate the properties of RB-SiC. The main stage of this process is the volatilization and pyrolysis of PR [19]. According to the TG curve of PR shown in Figure 2, the temperature for debinding was set to 500 °C. The heating rate was 0.5 °C/min from 400 to 500 °C. At the same time, in order to avoid defects during the debinding process, heat preservation was carried out at 450 °C for 30 min. In order to make sure that the green body has sufficient strength in the subsequent process, the pre-sintering was carried out immediately after the debinding, and the temperature of pre-sintering was 1650 °C [20]. Finally, the C/SiC preform was prepared, named P0. The program of debinding and pre-sintering are shown in Figure 3a.

(3) For impregnation and carbonization of PF solution

The impregnation and carbonization of carbon-source precursors is the key step to regulating the comprehensive performance of SiC ceramics. The PF solution was composed of PF (665) and anhydrous ethanol (Analytical Reagent, Beijing Institute of Chemical Reagents, Beijing, China) according to the mass ratio of 4:6. PF and anhydrous ethanol were purchased from Shengda Insulation Material (Changchun, China) and Institute of Chemical Reagents (Beijing, China), respectively. Theoretically, the carbon content in C/SiC preforms can be increased by multiple cycles of impregnation/carbonization cycles [16]. The TG curve of PF is shown in Figure 2. At 1000 °C, the curve is completely stable. Therefore, after impregnating P0 with PF solution for 12 h, carbonization was carried out at 1000 °C in a vacuum. Figure 3b shows the heating program for carbonization. The carbonized specimens were obtained when the furnace chamber was at room temperature, named P1. After two and three times of impregnation/carbonization of P0, P2 and P3 were obtained, respectively.

(4) For liquid silicon infiltration

LSI is the last step to regulate the performance of SiC ceramics in this experiment. As shown in Figure 1, commercial Si powders with a median diameter of 5 μm were placed on the surface of the C/SiC preform, and LSI was carried out at 1600 °C in a vacuum by using a chamber furnace. The heating program of LSI is shown in Figure 3c. Finally, the specimens of RB-SiC were obtained after LSI, named S0, S1, S2 and S3, corresponding to P0, P1, P2 and P3, respectively.

(5) Testing and characterization

The microstructures were observed by using scanning electron microscope (Phenom ProX, SEM, Phenom-World, Eindhoven, The Netherlands) and metallurgical microscopy (BX51 M, Olympus Corporation, Tokyo, Japan). The mass of P0, P1, P2 and P3 was weighed by an analytical balance (PTX-FA1004, Fuzhou Huazhi Scientific Instrument Co., Ltd., Fuzhou, China), and the density of P0, P1, P2 and P3 was determined by the weight and volume of the specimens [21].

In order to characterize the content of carbon in C/SiC, the weighting method and TG method were performed. The weighting method was used to oxidize C/SiC in muffle furnace (TCXC1200, Shanghai tong ke electric furnace Equipment Co., Ltd., Shanghai, China) under-air conditioning, and the TG method was used to oxidize C/SiC under air-conditioning, with a thermal analyzer (DSC, STA449 F3, Netzsch, Free State of Bavaria, Germany). The temperature range was from room temperature to 1000 °C. At the same time, SiC powders were used in both methods to eliminate the error caused by the oxidation of SiC. Both results above were calculated by the mass changes before and after oxidation.

The apparent porosity and bulk density(ρSiC) of RB-SiC were measured according to Archimedes principle. An X-ray diffractometer (XRD, Bruker/D8FOCUS, Bruker Instrument Co., Ltd., Karlsruhe, Germany), which was equipped with a Cu Kα radiation source, was used to obtain the phase composition of specimens. The Raman spectrometer (HOOKE P300, HOOKE Instruments Ltd., Changchun, China) was used for Raman measurements.

As for the thermal dimensional stability, a thermomechanical analyzer (DIL 402C, NETZSCH, Free State of Bavaria, Germany) was used to measure the linear expansion coefficient (CTE) of the RB-SiC. The temperature of the test was 0 to 100 °C, with a heating rate of 10 °C/min. The thermal diffusivity(α) of the RB-SiC was measured by LFA467Hyper Flash (NETZSCH, Selb, Germany). The specific heat capacity (cp) of the RB-SiC was measured by differential scanning calorimetry (DSC200F3, NETZSCH, Selb, Germany) from −30 to 20 °C, under an argon atmosphere. Finally, the thermal conductivity (λ) of S0, S1, S2 and S3 was calculated according to Equation (1) [22]:

(1) λ=α·ρSiC·cp

**Figure 4:**
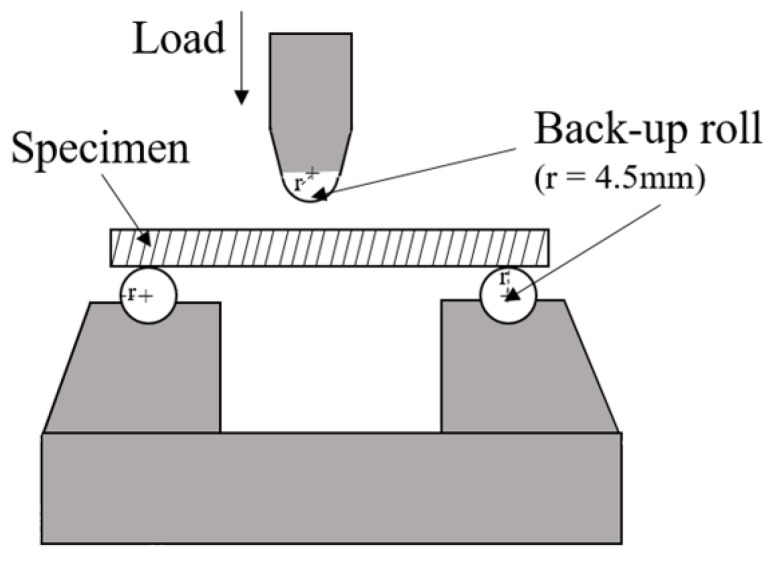
Schematic diagram of three-point bending test.

As for the mechanical strength of the specimens, the three-point bending method was performed by using a universal testing machine equipped with a grating micrometer (TG115, Chengdu Yuanheng precision measure and control technology Co., Ltd., Chengdu, China). The tested span is 30 mm and the loading rate is 0.5 mm/min. The size of specimens was 36 mm × 4 mm × 3 mm. The schematic diagram of the mechanical strength of the specimens is shown in Figure 4.

## 3. Results and Discussion

Generally, the macroscopic properties of RB-SiC depend on their microstructure, and the microstructure of RB-SiC is closely related to that of the C/SiC preform. Therefore, this section focuses on analyzing the evolution of the microstructure and properties of C/SiC preform and RB-SiC, respectively. The effects of cyclic impregnation/carbonization of the carbon precursor on the characteristics of C/SiC preform and RB-SiC are summarized.

### 3.1. Characterization of C/SiC Preform

**Figure 5:**
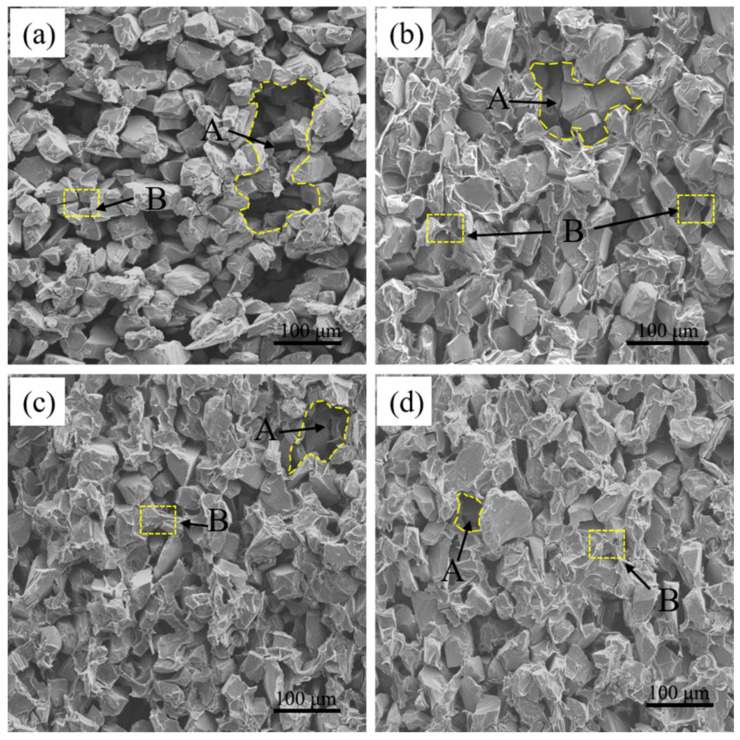
Microstructure of the specimens: (**a**) P0, (**b**) P1, (**c**) P2 and (**d**) P3. (Area A represents the pores and Area B represents the connection among SiC particles.)

Figure 5 shows the microstructure of P0, P1, P2 and P3. It can be seen that the carbonized body is composed of carbon, SiC and pores. In Figure 5, Area A represents the pores. Assuming the phenomenon of capillary channel blockage does not occur, it is well-known that Area A will be filled with secondary SiC (β-SiC) and residual Si after the LSI process. The β-SiC is formed by the reaction of Si and carbon. If Area A is larger, the size of residual Si is larger in the RB-SiC, and this will deteriorate the properties of the ceramics. Therefore, it is necessary to reduce the size of Area A to improve the properties of RB-SiC. As shown in Figure 5, by comparing P0, P1, P2 and P3, it can be found that the number and size of Area A are significantly reduced after cyclic impregnation/carbonization of PF solution. The results indicated that the content and size of residual Si in RB-SiC would be effectively reduced and the content of β-SiC would be increased after LSI process. The microstructure of RB-SiC would be improved theoretically. In addition, Area B represents the connection among SiC particles, as shown in Figure 5. If the connections among the SiC particles are weak, the C/SiC preform can be easily damaged during the experiment, and this has a negative effect on the properties and dimensional accuracy of the RB-SiC. As can be seen from Figure 5a, SiC particles in P0 are stacked with each other with few connections. Figure 5b–d shows that the connection among SiC particles is gradually enhanced after multiple cycles of impregnation/carbonization of C/SiC preform with PF solution. Therefore, according to the analysis above, the multiple cycles of impregnation/carbonization of PF solution for the C/SiC preform can regulate the microstructure of the carbonized body, and this is beneficial to regulate the microstructure and properties of RB-SiC composites.

**Figure 6:**
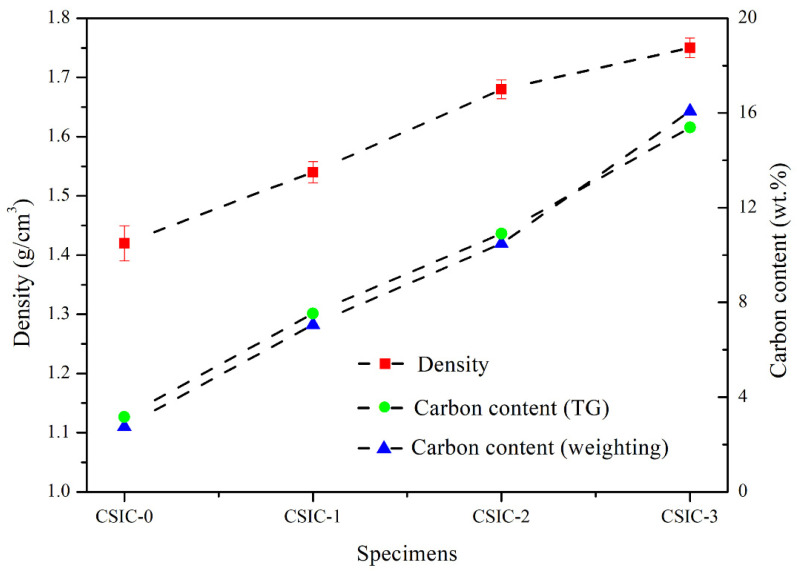
Density of C/SiC preform, carbon content determined by TG method and carbon content determined by weighting method.

As shown in Figure 6, the density of C/SiC preform increased gradually after cyclic impregnation/carbonization of PF solution. It shows that the content of carbon in the carbonized body was effectively increased by this method. The densities of P0, P1, P2 and P3 were 1.42, 1.54, 1.68 and 1.74 g/cm^3^, respectively. The density of carbonized bodies increased by 8.45%, 9.09% and 4.17% during the three cycles of impregnation/carbonization with PF solution. This is due to the highest porosity of P0, which is conducive to the impregnation of PF solution. After a cycle of impregnation/carbonization, the pyrolytic carbon formed a reticulated structure inside the C/SiC preform. The reticulated structure provided additional attachment points for the PF solution. Therefore, the density of C/SiC preform still has a high growth rate after cyclic impregnation/carbonization is performed twice. However, the density of P3 increased by only 4.17% compared with P2, indicating that it is difficult to significantly increase the content of carbon in the C/SiC preform by the same process [16]. The calculation results of the content of carbon in C/SiC by using the weighting method and TG method are shown in Figure 6. Although the weighting method is not as accurate as the TG method, the results of the two methods are similar for P0, P1 and P2. The difference between the two methods is the size of specimens. The specimens required by the TG method are 3 to 5 mg, while the specimens used by the weighting method are about 100 g. Therefore, without considering the errors, the weighting method could account for the carbon content of the whole specimen, while the TG method represented the carbon content of the local specimen. If the composition of the specimens was uniform, the results calculated by the two methods above should be similar. As can be seen from Figure 6, the carbon content of P3 calculated by the two methods is slightly different. This may be due to the carbon segregation phenomenon in the specimens after cyclic impregnation/carbonization for three times.

In some cases, when the carbon content of C/SiC preforms is supersaturated, the phenomenon of capillary channel blockage can be found in RB-SiC, resulting in the presence of residual carbon in RB-SiC. The residual carbon is detrimental to the properties of RB-SiC [23]. Therefore, the theoretical optimal value of carbon content in C/SiC preforms was calculated by using Equation (2) [16]:

(2) $$\mu =(1-{[1+\frac{Mc}{Msic}\left(\frac{W-Vsic1}{Vsic1})\right]}^{-1})\cdot 100\%$$

where μ (wt.%) is the maximum carbon content in C/SiC preform (avoiding the phenomenon of capillary channel blockage); Mc and Msic are the molar masses(g/mol) of carbon and SiC, respectively; Vsic1 (vol.%) is the volume fraction of SiC in the C/SiC preform; and W is the volume ratio of specimens before and after LSI. After 3D printing, the content of SiC in the green body was 47 vol.%. During the debinding/pre-sintering process, the volume shrinkage of the specimens was about 10% and the volume did not change after cyclic impregnation/carbonization of PF solution. In addition, the volume of specimens did not change before and after LSI process [18]. Therefore, Vsic1 is 52.22 vol.% and W is 100%. We substituted the values of Vsic1, W, Mc (12 g/mol) and Msic (40 g/mol) into Equation (2) to calculate the value of μ, which is 21.54 wt.%. As can be seen from Figure 6, the carbon content of C/SiC (including P0, P1, P2 and P3) is not supersaturated, so it can theoretically avoid the phenomenon of capillary channel blockage.

**Figure 7:**
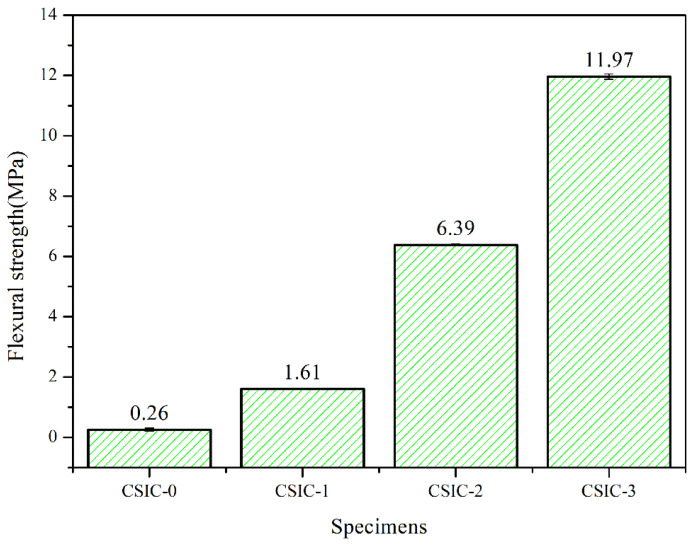
Flexural strength of P0, P1, P2 and P3.

**Figure 8:**
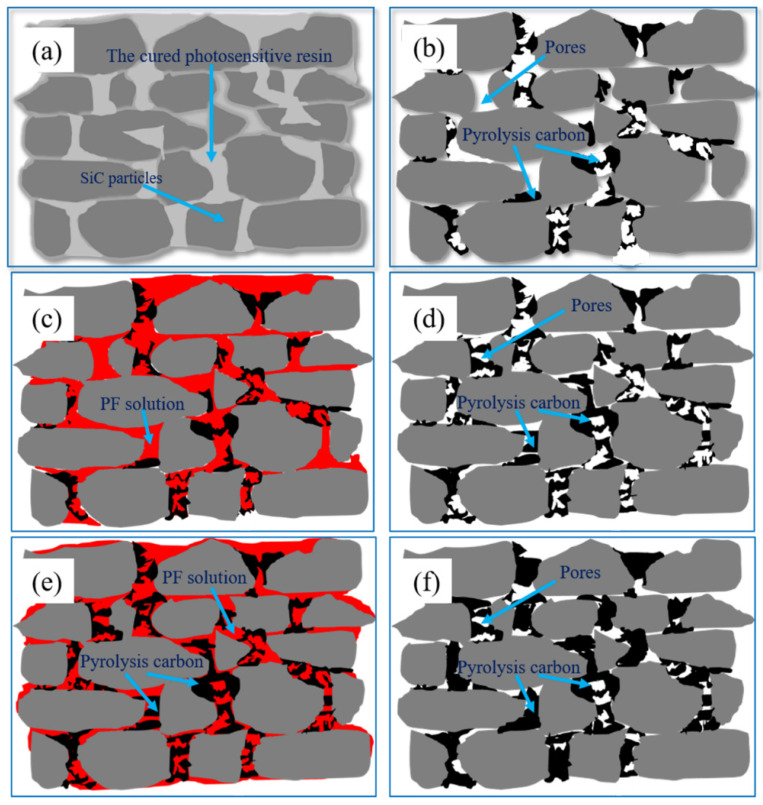
Schematic diagram of microstructure in C/SiC preforms during cyclic impregnation/carbonization of PF solution: (**a**) the green body after 3D printing, (**b**) P0, (**c**) the first impregnation of PF solution for P0, (**d**) P1, (**e**) the first impregnation of PF solution for P1 and (**f**) P2.

Figure 7 shows the flexural strength of P0, P1, P2 and P3. In the C/SiC preform, the SiC particles are connected by pyrolytic carbon, so its strength has an important relationship with the content and distribution of pyrolytic carbon. It can be seen from Figure 5a that P0 has more pores than other specimens. The pores would decrease the mechanical properties of C/SiC preform. Therefore, P0 has the lowest flexural strength among these specimens. During the process of manual handling before LSI, the C/SiC preform is prone to forming cracks, collapse or other defects affecting dimensional accuracy, due to the poor mechanical properties of the preform. As shown in Figure 7, the strength of the preform gradually increased after cyclic impregnation/carbonization, which would benefit the integrity and accuracy of the specimens during the subsequent operations. Figure 8 shows the schematic diagram of microstructure in C/SiC preforms during cyclic impregnation/carbonization of PF solution. It can be seen from Figure 2b that the carbon pyrolyzed by PR is less than 5 wt.%, and it can be found in Figure 8b that this part of carbon formed the weak connections among SiC particles, so P0 showed the lowest strength. After impregnation/carbonization of the PF solution for the first time, the carbon was not only on the surface of SiC but also among the SiC particles, which could play a role of connection, as shown in Figure 8b. The carbon pyrolyzed by PF made P1 stronger than P0. Compared Figure 8d with Figure 8f, the pyrolytic carbon in the C/SiC preform increased, and the connections among SiC particles became stronger. This is because, when the PF solution was impregnated for the second time, the PF could be attached not only to the surface of SiC particles, but also to the connections (formed by pyrolytic carbon) among the SiC particles in Figure 8d, as shown in Figure 8e. As a result, in addition to the layer of the pyrolytic carbon becoming thicker, the important thing was that the connections among the SiC particles became stronger, as shown in Figure 8f. This is the main reason for the improvement of the flexural strength of P2. Similarly, the flexural strength of P3 had been improved. In the LSI process, the connections formed by pyrolytic carbon are transformed to β-SiC. In addition to increasing the content of SiC, the size of residual Si is effectively reduced, which is beneficial to improve the performance of RB-SiC composites.

### 3.2. Characterization of RB-SiC

For space optical mirrors, in order to stabilize the focal length and obtain high-quality images, the mirrors should have excellent thermal dimensional stability and mechanical properties.

#### 3.2.1. Compositional Characteristics of RB-SiC Ceramics

**Figure 9:**
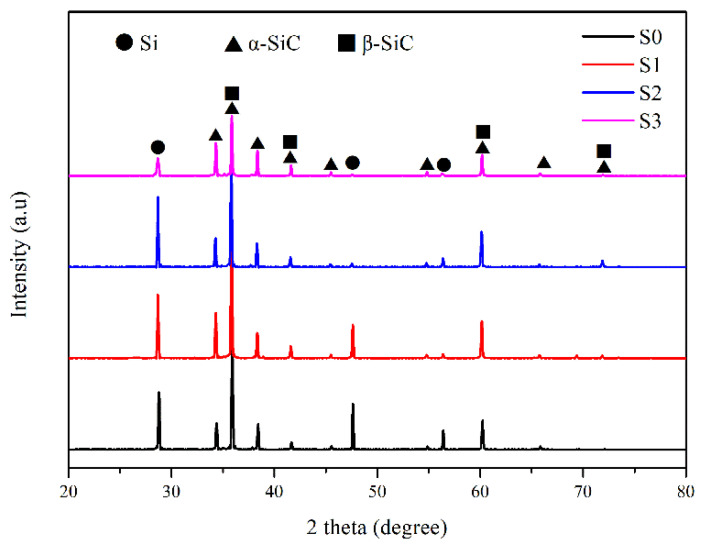
XRD pattern of S0, S1, S2 and S3.

Figure 9 shows the XRD analysis results of all the sintered bodies. The results show that all the sintered bodies are composed of Si, raw α-SiC and reaction-formed β-SiC. The presence of crystalline Si is due to excess Si during LSI process. After liquid Si infiltrated into the pores of C/SiC preform, part of Si reacted with carbon and the residual Si filled the pores [23]. With the increase of impregnation/carbonization cycles, the Si content gradually decreased, as was clearly indicated by the strongest diffraction peaks of Si, from crystal planes (111), (220) and (311) at 2θ of 28.4°, 47.3° and 56.1°, respectively. However, the content of β-SiC could not be marked by XRD patterns. On the one hand, the diffraction peaks of β-SiC are overlapped with α-SiC. On the other hand, the reaction of Si and carbon leads to exothermic phenomena of about 500 K [24], and the temperature of this experiment is 1873 K, which can transform β-SiC to α-SiC [24,25].

**Figure 10:**
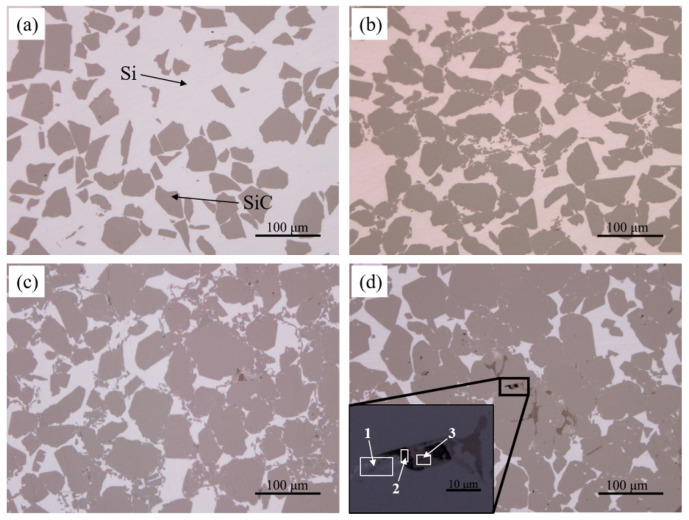
Morphology of the polished surface of RB-SiC for (**a**) S0, (**b**) S1, (**c**) S2 and (**d**) S3.

As shown in Figure 10, the content and distribution of each phase in the RB-SiC can be more intuitively observed after grinding and polishing. The light is Si, and the dark is SiC. Compared with S0 to S3, with the increase of impregnation/carbonization cycles, more SiC was formed in the final body, while the content and size of the residual Si were decreased obviously. Compared with S0, S2 had many fine SiC particles, which were connected with the surrounding large SiC particles in a reticulated structure. This is due to the thick connections among SiC formed by the pyrolytic carbon in P2. When molten Si infiltrated the pores and reacted with carbon, the secondary SiC retained this structure and the reticulated structure of SiC could effectively separate the residual Si. It shows that the content and size of Si in RB-SiC are effectively optimized after two cycles of impregnation/carbonization of PF solution, as shown in Figure 10c. However, different regions could be observed in S3 from S0, S1 and S2. In addition to the light (Si) and the dark (SiC), Area 1, Area 2 and Area 3 were observed at high magnification in S3. The XRD results showed no difference between S3 and other specimens, as shown in Figure 9. The limitation of the XRD method is that it has no obvious diffraction peaks for the amorphous materials [26]. Therefore, in order to further analyze the composition of S3, Raman spectrum analysis was carried out because of its sensitivity to both crystalline materials and amorphous materials [26].

**Figure 11:**
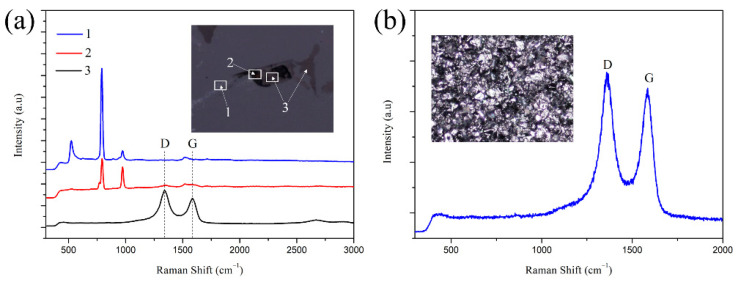
Raman analysis of (**a**) S3 and (**b**) pyrolytic carbon produced by PF.

Raman spectrum analysis was performed, on S3 and the results are shown in Figure 11. As known. The characteristic peak of SiC is located at 790 and 960 cm^−1^, and the characteristic peak located at 520 cm^−1^ is typical of Si [27]. Therefore, Area 1 consists of Si and SiC, while Area 2 consists of only SiC. However, the SiC shown at Area 2 appeared black under the optical microscope, and this is significantly different from the SiC (the color is dark) in S0, as shown in Figure 10. As reported by Q. Wang [28] and A. Inam [29], the formation of two characteristic bands, located at 1350 cm^−1^ (D peak) and 1590 cm^−1^ (G peak) in the Raman spectrum, indicates the presence of carbon. The intensity of the D peak represents the poor crystalline part of carbon and the intensity of the G peak represents the crystalline part of carbon. The crystallinity of carbon is derived from the intensity ratio of the D peak to the G peak [28]. Table 1 shows the Raman analysis results of S3 and pyrolytic carbon. By calculation, the ratio of D peak to G peak in S3 and pyrolytic carbon is 1.29 and 1.09, respectively. According to the result of A. Inam [29], the ratio of D peak to G peak ranges from 1.0 to 2.6, indicating poor crystallinity of carbon, namely amorphous carbon. It indicated that part of pyrolytic carbon failed to react with liquid Si after three cycles of impregnation/carbonization of PF solution, and the residual carbon existed in S3 as the impurities. The residual carbon was easy to peel off during the machining process, resulting in holes on the surface of the specimens for S3. When S3 was tested by Raman spectrum, the laser beam passed through the hole and irradiated on the SiC under the hole. At the same time, this could also explain the reason why the color of SiC at Area 2 was different from the color of SiC in S0, as shown in Figure 10.

**Figure 12:**
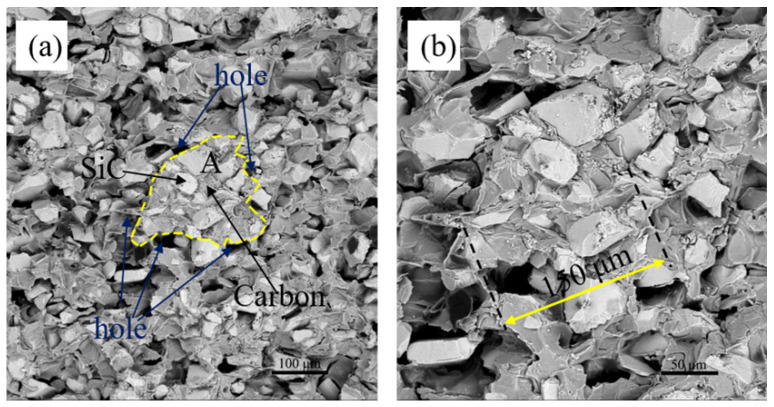
Back-scattered electron (BSE) images of microstructure of (**a**) P3, and (**b**) magnification of the region marked by yellow line of dashes.

As discussed above, it can be inferred that the typical phenomenon of capillary channel blockage existed in S3. However, according to the calculation results of Equation (2), the carbon content in P3 had not reached the theoretical value to cause the phenomenon of capillary channel blockage. In order to understand the specific causes of this phenomenon, it is necessary to observe the microstructure of P3 again. The P3 was composed of SiC as the bright and carbon as the dark gray, as shown in Figure 11. The phenomenon of carbon segregation was observed in Area A in Figure 12a. Area A consisted of SiC and carbon, with a few holes in the plane. In P3, the holes were the channel for the infiltration of liquid Si. During the LSI process, the liquid Si reacted with the carbon at the edge of Area A to form β-SiC. Fu [30] reported that the newly formed SiC acted as a barrier to the further reaction of the carbon in the core of are A with liquid Si around Area A. The newly formed SiC gradually increased by the diffusion-controlled mechanism [31]. Jesse C. Margiotta [31] and M.H. Hon [32] revealed that the diffusion rate of carbon in β-SiC was three orders of magnitude higher than that of Si in β-SiC. It showed that the reaction of carbon and Si depended on the dissolution and diffusion of carbon in β-SiC. The thickness, τ, of dissolved carbon can be calculated by using the following equation [33].

(3) $$\tau \approx \sqrt{De\cdot t}$$

where *t* is the reaction time (*t* = 2 h), and *D_e_* is the effective diffusion coefficient of carbon in SiC, which is given by the Arrhenius equation.

(4) $$D_{e}=D_{0}\cdot \exp(\frac{-Q}{RT})$$

where D_0_ is pre-exponential factor (D_0_ = 2 × 10^−6^ cm^2^/s), *Q* is activation energy (*Q* = 132 KJ/mol), *R* is the gas equilibrium constant (*R* = 8.314 J/(mol·K)) and *T* is the temperature of LSI process (*T* = 1873 K). According to Equations (3) and (4), τ was calculated to be 17.33 μm. As shown in Figure 12b, the maximum diameter of Area A is about 150 μm. Therefore, in the case of insufficient liquid Si in the direction perpendicular to Area A, the carbon in the core of Area A would not react with Si, which was easy to lead to the phenomenon of capillary channel blockage. For example, it is assumed that the shape of carbon in the C/SiC preform is spherical. When the diameter of carbon is greater than 34.66 μm, the residual carbon would be observed in RB-SiC after LSI, as shown in Figure 10d.

#### 3.2.2. Comprehensive Performance of RB-SiC Ceramics

**Figure 13:**
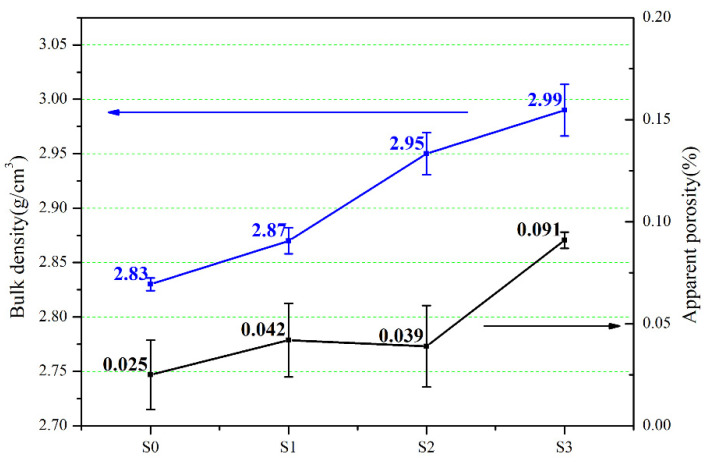
Bulk density and apparent porosity of S0, S1, S2 and S3.

As known, the density of SiC (3.21 g/cm^3^) is greater than that of Si (2.33 g/cm^3^). Therefore, the bulk density of SiC increases, indicating that the content of SiC increases. Although residual carbon existed in S3, the content of SiC in S3 was still the highest, as can be seen from the results of bulk density in Figure 13. At the same time, Figure 13 shows the results of the apparent porosity of RB-SiC measured by the Archimedes method. The results show that the apparent porosity of S3 is significantly higher than that of other specimens. This is the fact that the residual carbon on the surface of the specimens is easy to peel off during the machining process, leading to the increase of apparent porosity for S3.

**Figure 14:**
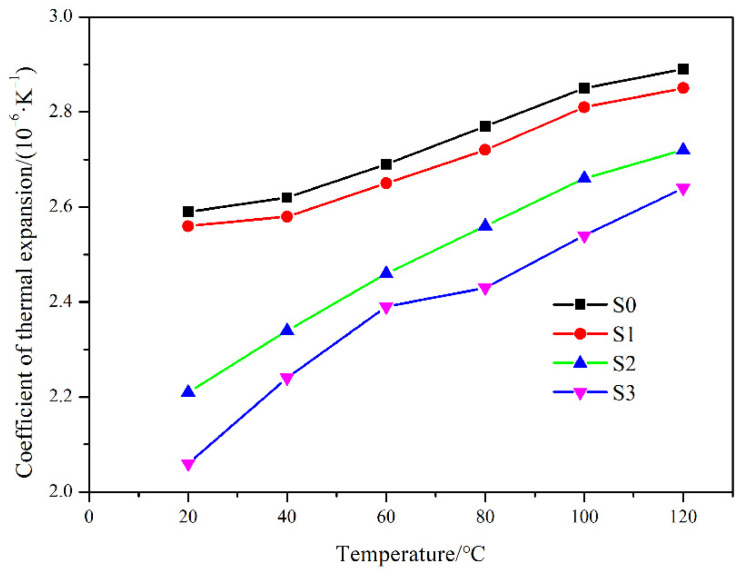
Coefficient of thermal expansion from 0 °C to each temperature range for S0, S1, S2 and S3.

Figure 14 shows the coefficient of thermal expansion (CTE) of RB-SiC as a function of temperature. Similar to polycrystalline materials, the CTE of RB-SiC varies with temperature and usually increases with an increasing temperature [34]. With the increase of impregnation/carbonization cycles, the CTE of RB-SiC decreased at all temperature points, as shown in Figure 14. According to the results of H. Watanabe [35] and A.L. Marshall [36], the CTE of Si and SiC is 2.6 × 10^−6^ K^−1^ and 2.3 × 10^−6^ K^−1^ at room temperature, respectively. Therefore, the CTE of RB-SiC decreased as the content of Si in the RB-SiC composites decreased. In addition, it can be found that the CTE of S0 and S1 has the same variation trend, which is different from S2 and S3. Raman spectrum showed that the residual carbon existed in S3. According to the report of J.F. White [37], the CTE of carbon is 2.1 × 10^−6^ K^−1^ at room temperature, so this is another reason to explain the lowest value of CTE for S3. The existence of residual carbon as the impurity and pores led to an inconsistent trend of curve changes with S0 and S1. The reason for the inconsistent trend of S2 and S1 curves might be that S2 also had a small amount of amorphous carbon. However, because the content of amorphous carbon was low, it could not be observed under the optical microscope mode.

**Figure 15:**
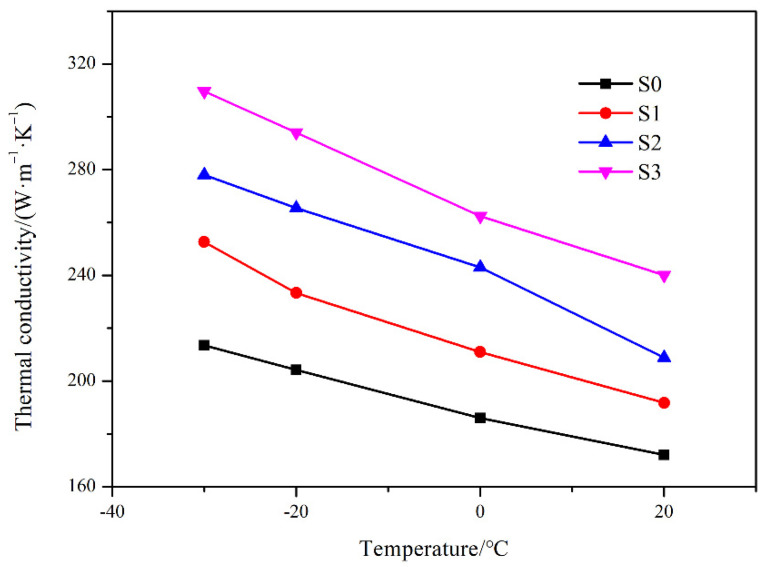
Thermal conductivity of S0, S1, S2 and S3 as function of temperature (solid line is empirical formula fitting to data).

Besides CTE, thermal conductivity is also one of the key factors to ensure thermal dimensional stability. The thermal conductivity was obtained according to Equation (1), as shown in Figure 15. Usually, when the temperature increases, the thermal conductivity of the ceramics decreases [30]. When comparing S0 with S1, S2 and S3, we see that the thermal conductivity was improved via cyclic impregnation/carbonization of PF solution. In general, the thermal conductivity of materials is affected by defects, grain boundaries and composition of materials. In contrast to the heat transfer of free electrons in metals, the heat transfer of ceramics is mainly determined by lattice vibration [30]. The existence of defects and grain boundaries will interfere with lattice vibration and lead to the reduction of the thermal conductivity of ceramics. As reported by Li [38] and Du [39], the thermal conductivity of SiC and Si was 490 and 145 W/(m·K), respectively. From the analysis above, it can be seen that the defects and the content of SiC are the highest in S3, but the thermal conductivity of S3 is still the highest, indicating that the thermal conductivity is primitively determined by the composition of RB-SiC in this study. Therefore, the CTE of RB-SiC could be reduced and the thermal conductivity of RB-SiC could be increased by cyclic impregnation/carbonization of PF solution for C/SiC preform. This method improved the thermal dimensional stability of RB-SiC composites.

The strength of RB-SiC mainly depends on the following [40,41]:

(1) The composition and distribution of phases;

(2) Interconnectivity of the SiC in the RB-SiC;

(3) Porosities.

**Figure 16:**
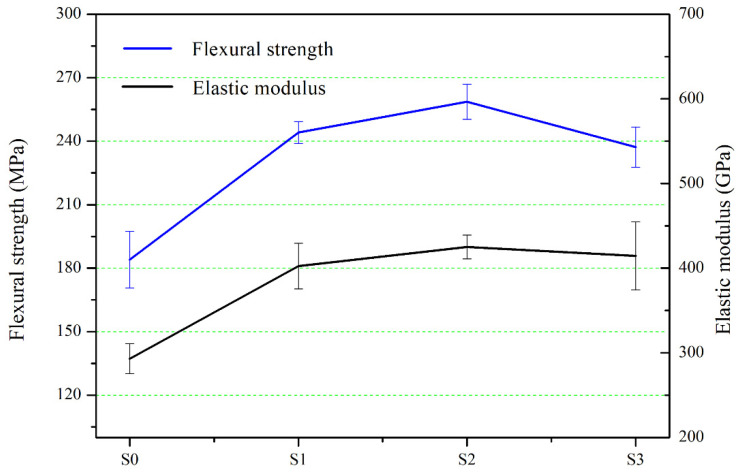
Flexural strength and elastic modulus of S0, S1, S2 and S3.

As discussed, RB-SiC is composed of SiC and Si; the latter has the lower mechanical properties. The content of Si in RB-SiC was decreased with the increase of impregnation/carbonization cycles. Figure 16 shows the mechanical properties of S0, S1, S2 and S3. The flexural strength and elastic modulus of S0 are 183.99 ± 13.45 MPa and 230.45 ± 5.3 GPa, respectively. As can be seen from Figure 10c, S2 had the highest degree of interconnectivity and content of the SiC compared with S0 and S1. Therefore, S2 obtained a higher strength compared with S0 and S1, and the flexural strength and elastic modulus of S2 are 258.63 ± 8.33 MPa and 425.16 ± 14.15 GPa, respectively. As for S3, the mechanical properties curve showed a downward trend. Due to the existence of residual carbon in S3, the strength of S3 was reduced, as shown in Figure 16. The mechanical properties of amorphous carbon are inferior to SiC and Si, and the amorphous carbon existed in S3 as the impurity to deteriorate the mechanical properties of S3. In addition to composition, cracks, holes and other defects could also deteriorate the properties of materials. The residual carbon on the surface of specimens was easy to peel off during the machining process, thus increasing the porosity of the materials. When the load was applied to these defects, the source of crack propagation was easily formed at the defects, thus leading to the fracture of the materials and reduction of the strength of the materials. In the light of the above, when the C/SiC preform was impregnated/carbonized with PF solution twice, the RB-SiC obtained excellent comprehensive properties. Table 2 shows the comparison of the flexural strength of SiC prepared by SLA and LSI. Tian [10] mixed the PF with the PR solution, but this method resulted in an increase in the viscosity of slurry, and this was not conducive to SLA process. In this paper, the value of S2’s flexural strength was close to that of Zhang [15]. However, PF has the advantages of low cost and simple operation compared with carbon fiber. At present, the space optical mirrors in orbit were prepared by traditional manufacturing techniques. Therefore, Table 3 shows the mechanical properties comparison of SiC prepared by using SLA, with traditional manufacturing techniques. It can be seen that the flexural strength of SiC ceramics prepared by this method is close to that of the traditional method and has higher specific stiffness.

## 4. Conclusions

The RB-SiC was prepared via SLA and LSI. The properties of the RB-SiC were improved via cyclic impregnation/carbonization of the C/SiC preforms, with carbon as the precursor. This is an effective method to regulate the microstructure of C/SiC preform and optimize the composition and distribution of RB-SiC, and the comprehensive performance of RB-SiC was improved. The specific conclusions are as follows:

(1) Microstructure of C/SiC preform

The microstructures of the C/SiC preform were regulated by cyclic impregnation/carbonization of PF solution. The results showed that the pores in C/SiC preform became smaller and uniform with the increase of cyclic impregnation/carbonization of PF solution, and the connection between SiC particles was enhanced.

(2) Microstructure of RB-SiC composites

This method could decrease the content of Si in RB-SiC composites. The results showed that, when the impregnation/carbonization cycle was performed twice, the reticulated structure of SiC appeared in RB-SiC, which effectively reduced the size of residual Si.

(3) Comprehensive properties of RB-SiC

RB-SiC had the best comprehensive properties after the impregnation/carbonization of C/SiC preform with the PF solution being performed twice. The results showed that the flexural strength, bulk density and elastic modulus of S2 were 258.63 ± 8.33 MPa, 2.95 ± 0.02 g/cm^3^ and 425.16 ± 14.15 GPa, respectively. In addition, thermal dimensional stability of RB-SiC was also improved by this method.

According to the analysis above, it is a simple and effective method to regulate the properties of SiC prepared by SLA and LSI, especially when the C/SiC preforms are impregnated/carbonized with PF solution for twice. The properties of RB-SiC prepared by this method are close to those prepared by traditional methods, and this proves that SLA combined with LSI technology has the potential to prepare SiC spatial optical mirrors with complex structures and excellent properties.

## Figures and Tables

**Table 1:** Intensities and wave numbers(cm^−1^) corresponding to D and G peaks of the Raman spectra of specimen S3 and pyrolytic carbon in Figure 10.

Specimens	Band	Max Intensity (a.u)	Wave Number (cm^−1^)
S3	D	17,779	1341.98
	G	13,823	1582.80
Pyrolytic carbon	D	3778	1358.52
	G	3465	1585.35

**Table 2:** Flexural-strength comparison of SiC prepared by SLA and LSI with reported works from the literature.

Reference	[10]	[12]	[15]	[17]	This work
3D printing	SLA	SLA	SLA	SLA	SLA
Main raw materials of 3D printing	PF + PR + SiC	PR + SiC	C_f_ + PR + SiC *	PF + PR	PR + SiC
After debinding	C/SiC	C/SiC	C/C_f_/SiC	Porous carbon preforms	C/SiC
Other process	No	No	No	No	Cyclic impregnation/carbonization of PF solution in C/SiC preform
Sintering process	LSI	LSI	LSI	LSI	LSI
Final body	Si/SiC	Si/SiC	Si/SiC	Si/SiC	Si/SiC
Flexural strength	127.80	210.40	262.60	239.00	258.63

* C_f_ represents the carbon fiber.

**Table 3:** Comparison of mechanical properties of SiC prepared by SLA with traditional manufacturing techniques.

Fabrication Process	Flexural Strength/MPa	Elastic Modulus (E)/GPa	Density(ρ)/(g/cm^3^)	Specific Stability/(E/ρ)	Reference
Preferred Value	Large	Large	Small	Large	
Gelcasting + LSI	300 ± 20	——	3.04	——	[5]
Powder injection molding + LSI	225	427	3.14 ± 0.02	135.99	[16]
Powder injection molding + LSI	——	318.7	3.04 ± 0.01	104.84	[42]
Slip casting + LSI	274 ± 15	——	3.01	——	[4]
Gelcasting + gas silicon infiltration	245	220	——	——	[43]
SLA + LSI	258.63 ± 8.33	425.16 ± 14.15	2.95 ± 0.02	144.12	This work

## Data Availability

The data presented in this study are available on request from the corresponding author.
